# Application of Diffusion Tensor Imaging Parameters to Detect Change in Longitudinal Studies in Cerebral Small Vessel Disease

**DOI:** 10.1371/journal.pone.0147836

**Published:** 2016-01-25

**Authors:** Eva Anna Zeestraten, Philip Benjamin, Christian Lambert, Andrew John Lawrence, Owen Alan Williams, Robin Guy Morris, Thomas Richard Barrick, Hugh Stephen Markus

**Affiliations:** 1 Neuroscience Research Centre, Cardiovascular and Cell Sciences Research Institute, St George's, University of London, London, United Kingdom; 2 Department of Radiology, Charing Cross Hospital campus, Imperial College NHS trust, London, United Kingdom; 3 Stroke Research Group, Clinical Neurosciences, University of Cambridge, Cambridge, United Kingdom; 4 Department of Psychology, King's College Institute of Psychiatry, Psychology, and Neuroscience, London, United Kingdom; Fraunhofer Research Institution of Marine Biotechnology, GERMANY

## Abstract

Cerebral small vessel disease (SVD) is the major cause of vascular cognitive impairment, resulting in significant disability and reduced quality of life. Cognitive tests have been shown to be insensitive to change in longitudinal studies and, therefore, sensitive surrogate markers are needed to monitor disease progression and assess treatment effects in clinical trials. Diffusion tensor imaging (DTI) is thought to offer great potential in this regard. Sensitivity of the various parameters that can be derived from DTI is however unknown. We aimed to evaluate the differential sensitivity of DTI markers to detect SVD progression, and to estimate sample sizes required to assess therapeutic interventions aimed at halting decline based on DTI data. We investigated 99 patients with symptomatic SVD, defined as clinical lacunar syndrome with MRI confirmation of a corresponding infarct as well as confluent white matter hyperintensities over a 3 year follow-up period. We evaluated change in DTI histogram parameters using linear mixed effect models and calculated sample size estimates. Over a three-year follow-up period we observed a decline in fractional anisotropy and increase in diffusivity in white matter tissue and most parameters changed significantly. Mean diffusivity peak height was the most sensitive marker for SVD progression as it had the smallest sample size estimate. This suggests disease progression can be monitored sensitively using DTI histogram analysis and confirms DTI’s potential as surrogate marker for SVD.

## Introduction

Cerebral small vessel disease (SVD) causes about 20% of ischaemic stroke, and is the major cause of vascular cognitive impairment, including dementia. Whilst magnetic resonance imaging (MRI) techniques play a crucial role in diagnosis they are also being used increasingly to monitor disease progression and have been proposed as surrogate markers to assess the effects of therapeutic interventions. This latter use is of potential importance in detecting change given that cognitive testing has been shown to be relatively insensitive in measuring longitudinal decline [1,2].

Conventional MRI hallmarks of SVD include T2-white matter hyperintensities (WMH) and lacunes of presumed vascular origin. However, diffusion tensor imaging (DTI) has been shown to be a more sensitive measure of white matter (WM) damage in SVD detecting abnormalities not only within WMH but also in apparently normal appearing white matter (NAWM) [3,4]. In cross-sectional studies correlations between DTI parameters and levels of disability and/or cognition have been found in a number of SVD cohorts [4–9], and these correlations are stronger than those with T2 WMH volume [6,10]. To date, however, there is little longitudinal DTI data to determine the sensitivity of different DTI parameters to change over time. Determining the magnitude and variability of change over time is needed to determine the feasibility of using DTI as a biomarker, as well as to allow estimation of sample sizes needed to identify therapeutic efficacy in clinical trials.

Several quantitative measurements can be derived from DTI [11,12]. Mean diffusivity (MD) describes the overall extent of water diffusion, is orientation independent and is a marker of WM ultrastructure. Fractional anisotropy (FA) provides information on the directionality of the diffusion tensor and therefore the organisation of and damage to the ultrastructure determined by axonal fibre packing, degree of myelination and axon diameter [13]. Axial diffusivity (AD) is measured along the principal axis of the diffusion tensor and is considered a putative axonal damage marker; whereas radial diffusivity (RD) is measured perpendicular to the principal axis and thought to be sensitive to the degree of hindrance that diffusing water molecules experience due to the axonal membrane and myelin [14,15]. Analysing the various anisotropy and diffusivity measurements provides a range of information on the underlying WM tissue, but with varying reliabilities and sensitivities.

This prospective longitudinal study aimed to determine the sensitivity of different DTI parameters to WM damage over a period of 3 years in a clinically defined cohort of symptomatic SVD patients with MRI confirmation of a corresponding small subcortical infarct as well as confluent WMH. It also used the DTI data to evaluate sample sizes required for clinical trials to demonstrate treatment efficacy, and determine which DTI parameters provide the most statistical power to detect change in such studies.

## Methods

### 2.1 Patients

Patients presenting with symptomatic SVD were recruited as part of the St George’s Cognition and Neuroimaging in Stroke (SCANS) study [6] from stroke services in three local South London hospitals (St George’s Hospital, King’s College Hospital and St Thomas’ Hospital). Symptomatic SVD was defined as a clinical lacunar stroke syndrome [16] with a neuroanatomically corresponding small subcortical infract as well as confluent areas of WMH of presumed vascular origin on MRI graded ≥2 on the semi-quantitative Fazekas scale [17]. Exclusion criteria were: cardio-embolic sources of stroke, large vessel disease with >50% stenosis in intra- or extra-cranial arteries, other central nervous system disorders, major psychiatric disorders and any cause of white matter disease other than SVD. Patients were recruited at least 3 months after their last stroke to avoid the influence of any acute ischaemic effects on neuropsychological function (the latter analysed in a separate study). Ethical approval for the study was obtained from the Wandsworth Research Ethics Committee and all patients gave written informed consent.

**Fig 1:**
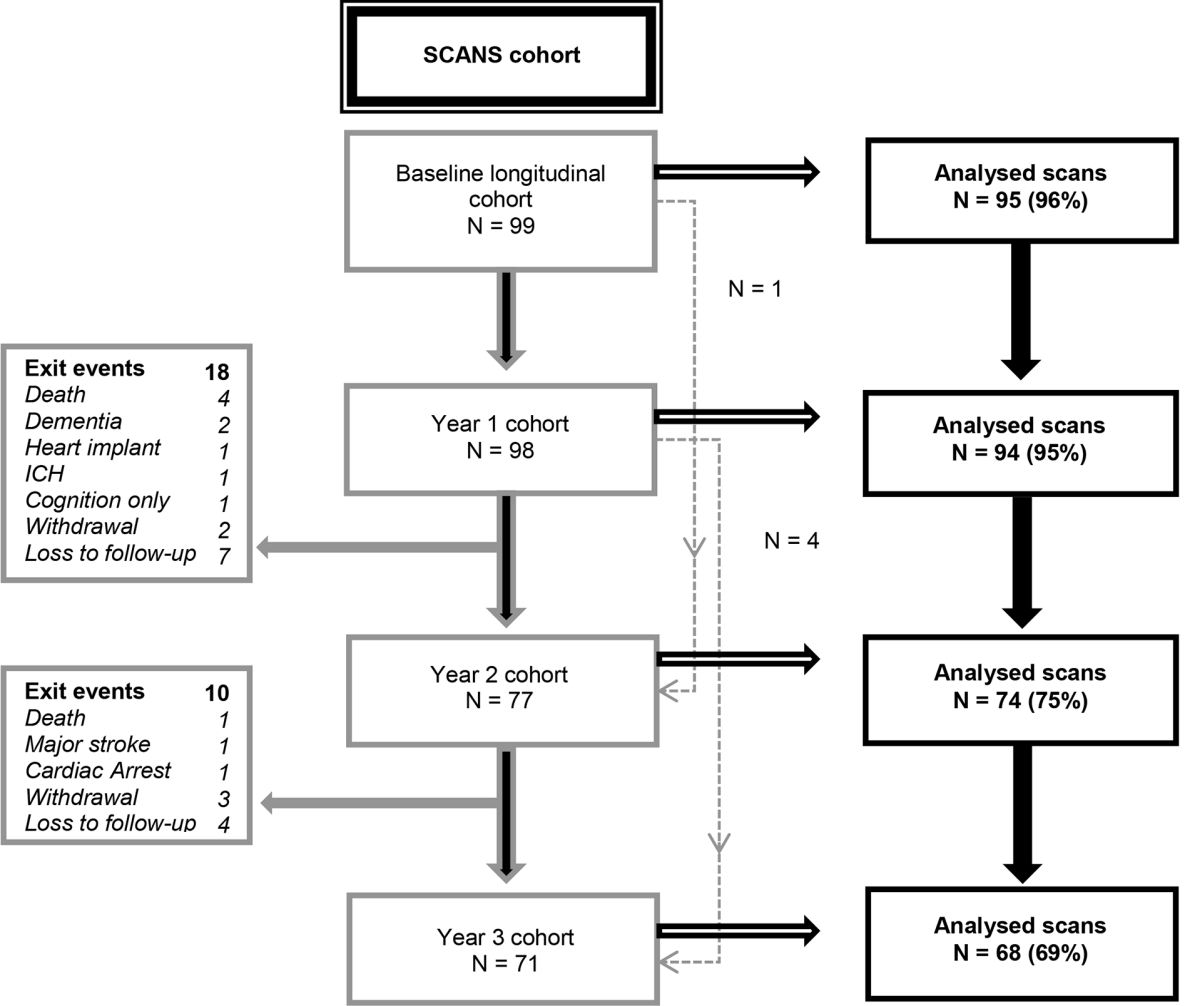
SCANS longitudinal imaging cohort. All available DTI scans without EPI warping were used in our analyses. Exit event coding: Death = patient passed away; Dementia = patient developed a primary dementia syndrome; Heart implant = patient was unable to do further MR sessions due to a heart implant; ICH = subject met exclusion criteria after intracerebral haemorrhage; Cognition only = patient withdrew from MR sessions but remained in study for cognition; Withdrawal = patient formally withdrew from the study; Loss to follow-up = patient was lost to follow-up; Major stroke = patient met exclusion criteria after major ischaemic stroke; Cardiac arrest = major cognitive impairment related to hypoxia was seen in a patient after a cardiac arrest.

One hundred and twenty one patients were recruited into the SCANS study [6,18,19] and of these, 99 completed one or more follow-up scanning sessions; reasons for exit of the other patients were death (7), declining further MR scanning (6), formally withdrawing from the total study (6), and being lost to follow-up (3) (see Fig 1 and missing data section for follow-up scanning frequencies). For this study DTI analyses were performed on the 99 patients with follow-up MRI scans available.

### 2.2 MRI acquisition protocol

MRI scans were performed using a 1.5-Tesla GE Signa HDxt system (General Electric, Milwaukee, WI, USA) with a maximum gradient amplitude of 33 mTm^-1^ and a proprietary head coil. Patients were neutrally positioned in the head coil using an alignment marker at the nasal bridge to standardise head position. Foam pads and a Velcro strap across the forehead were used to minimise head movement during the scan.

The imaging protocol lasted approximately 45 minutes and included the following scan sequences which all provided whole head coverage: I. axial Fluid Attenuated Inversion Recovery (FLAIR): repetition time (TR) = 9000ms, echo time (TE) = 130ms, inversion time (TI) = 2200ms, 28 slices of 5mm thickness without slice gap, field of view (FOV) = 240x240mm^2^, matrix = 256x192; II. coronal T1-weighted spoiled gradient recalled echo (SPGR): TR = 11.5ms, TE = 5ms, 176 slices without slice gap, FOV = 240x240mm^2^, matrix = 256x192, flip angle = 18°, providing 1.1mm^3^ isotropic voxels; and III. axial single shot spin echo planar diffusion-weighted imaging: TR = 15600ms, TE = 93.4ms, 55 slices without slice gap with isotropic voxels of 2.5mm^3^, FOV = 240x240 mm^2^, matrix = 96x96, 8 non-diffusion-weighted images (b = 0 smm^-2^) followed by diffusion-weighted volumes with diffusion gradients applied (b = 1000 smm^-2^) in 25 non-collinear directions and the negative of these.

### 2.3 MRI analysis

#### 2.3.1 Structural pre-processing and tissue segmentation

The steps used are detailed in Lambert et al [20], but are summarised below. Initially the T1-weighted and FLAIR images were rigidly aligned to MNI template space. Using SPM12 *New Segment* [21] the FLAIR and T1-weighted images were segmented into 1mm^3^ isotropic voxels, providing tissue class probability maps (TPMs) for WM, grey matter (GM), and cerebrospinal fluid (CSF). These segmentations were used to calculate deformation fields using a diffeomorphic registration algorithm (Shoot toolbox SPM12 [22]). Following skull stripping of the T1-weighted and FLAIR images, performed using the combined tissue segmentations at a threshold of 0.1, the T1-weighted and FLAIR images were warped to the group-average-template computed from this study cohort. A modified multivariate mixture of Gaussians SVD population specific TPMs [23] were generated to improve segmentation accuracy from the T1-weighted images for GM, WM and CSF. Additional TPMs for WMH were generated using the FLAIR and T1-weighted images together. SPM *New Segment* was subsequently re-run on the native space images using the population specific TPMs creating segmentation maps of GM, WM, CSF and WMH. To guarantee optimal correspondence of the maps with lesions as seen on the FLAIR image, WMH segmentation maps were optimised by binarisation at a manually set threshold for each patient by a blinded single rater. Manual refinement was performed where necessary to further improve accuracy. Reliability metrics were calculated as per Dunn et al [24]. The inter-rater reliability metrics were calculated using the mean values for each rater. WMH reliability metrics were checked using a subset of 20 randomly selected subjects by two raters (EAZ and CL). All potentially available time points were used for each subject in the reliability calculations. Both raters were blinded with respect to the subject identity and time point of each scan, and the scan order was randomized for each individual rater to avoid any potential bias. Intra-rater reliability metrics were: Standard error of mean = 247mm^3^, mean variability = 7.78% (standard deviation (SD) = 3.03%), Pearson’s intra-class correlation coefficient = 0.98. The inter-rater reliability metrics were: Standard error of mean = 187mm^3^, mean variability = 5.57% (SD = 3.99%), Pearson’s intraclass correlation coefficient = 0.99. Areas of lacunar damage were manually identified on native space T1-weighted images with reference to the FLAIR images, and segmented using a space-filling algorithm in ITK-SNAP (www.itksnap.org [25]). Grey scale intensity thresholds for the space-filling algorithm were set between 350 and 500 with 500 iterations. Manual refinement was required to ensure accurate representation of lacunes of presumed vascular origin adjacent to the lateral ventricles using ITK-SNAP. Reliability metrics were checked using a subset of 20 randomly selected scans by two raters (PB and CL), using the same procedure as the WMH reliability metrics. The intra-rater reliability metrics were: Standard error of mean = 3mm^3^, mean variability = 7.93% (SD = 4.89%), Pearson’s intra-class correlation coefficient = 0.99. The corresponding inter-rater reliability metrics were: Standard error of mean = 2mm^3^, mean variability = 4.32% (SD = 4.19%), Pearson’s intraclass correlation coefficient = 0.99.

**Fig 2:**
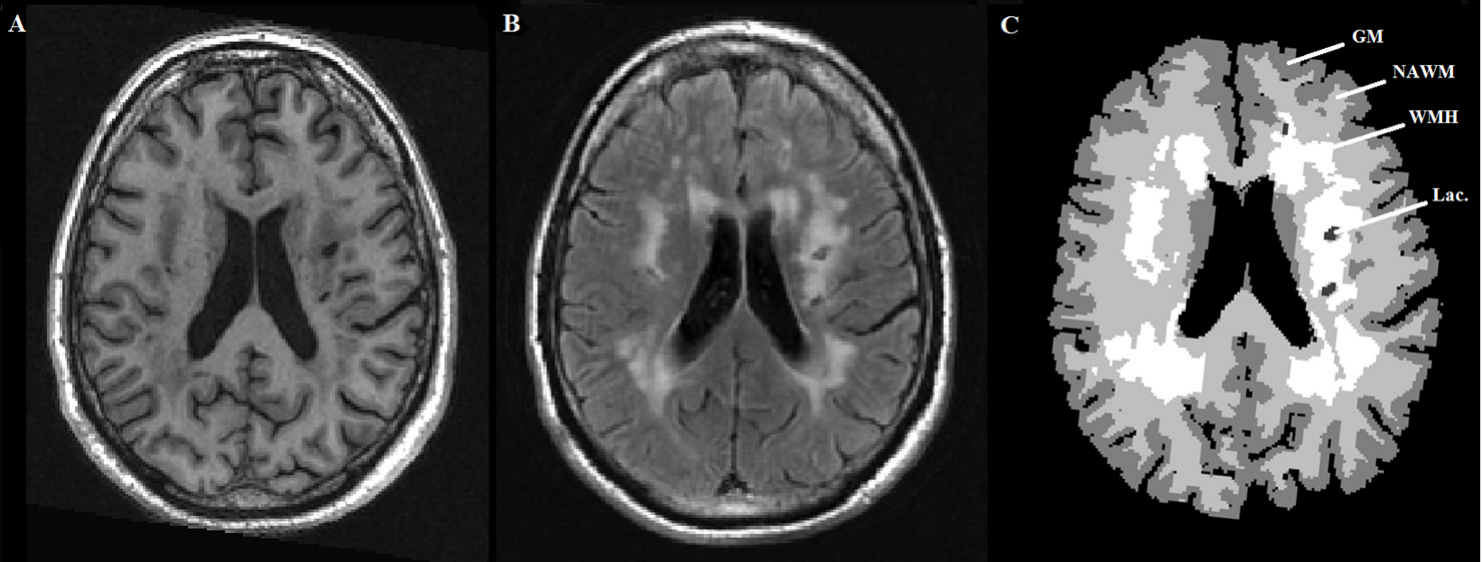
A. T1-weighted image B. FLAIR image C. Corresponding tissue segmentation map. Abbreviations: GM = grey matter; NAWM = normal appearing white matter; Lac. = lacune of presumed vascular origin; WMH = white matter hyperintensity.

The regions of lacunes of presumed vascular origin were removed from all tissue segmentation maps and classed as a fifth tissue class. All images were visually inspected to ensure consistent segmentation quality. The similar intensity profile of GM and WMH on T1-weighted images can cause tissue misclassification between GM and WM. To avoid this, the GM and WM maps were corrected using the binarised WMH and lacune of presumed vascular origin segmentations [20]. The NAWM tissue class was calculated by subtracting the WMH from the corrected WM map (Fig 2). GM, NAWM and WMH volumes were calculated in individual space by summing the corrected segmented areas. Total brain volume was defined as the sum of these three volumes.

#### 2.3.2 Diffusion pre-processing

Diffusion-weighted images were pre-processed to remove eddy current distortions using the FSL Linear Image Registration Tool (FLIRT, FMRIB Software Library, www.fmrib.ox.ac.uk/fsl [26]). The positive and negative diffusion gradient direction images were geometrically averaged to eliminate gradient cross-terms [27]. The 8 b = 0 images were averaged to give a T2-weighted echo planar image, throughout referred to as b0 images. Affine transformations, followed by non-linear transformations, were computed between intensity inverted, skull stripped T1-weighted images and the skull stripped b0 image using FMRIB Non-linear Image Registration Tool (FNIRT) [28]. The transformations were subsequently applied to the native T1-weighted images, masks and all tissue segmentations to obtain tissue class images in b0 space. The deformation quality was assessed for each individual using the warped T1-weighted and b0 images. Masks included voxels with probabilities of GM, NAWM or WMH >0.5. Spurious CSF voxels were removed from the tissue masks by application of a diffusivity threshold (i.e. voxels with MD >0.0026 mm^2^s^-1^ were considered to contain CSF and excluded).

#### 2.3.3 DTI histogram analysis

**Fig 3:**
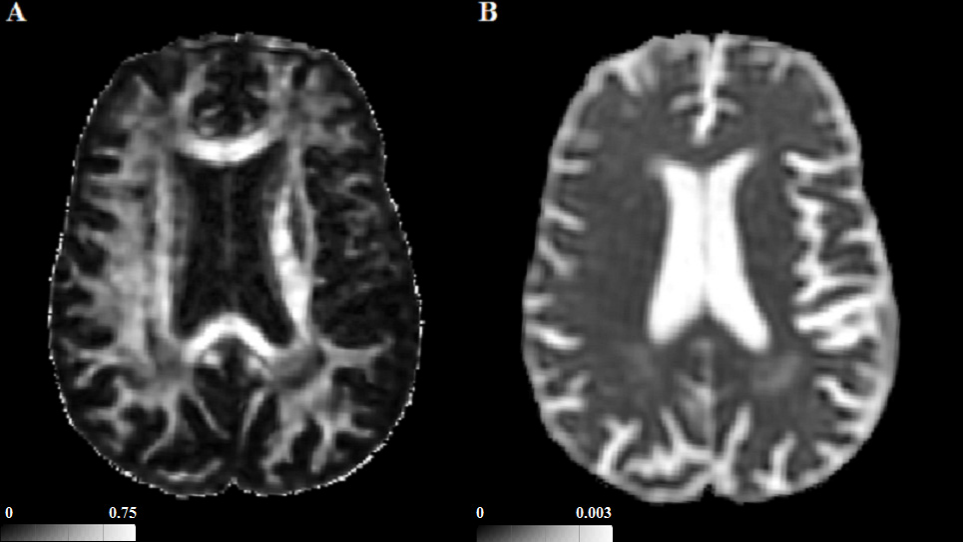
A. An axial fractional anisotropy and B. mean diffusivity map (mm^2^s^-1^). Images are from a 78 year old male from the SCANS cohort and representative of DTI-derived maps of symptomatic cerebral small vessel disease patients.

Using DTIfit in FSL a diffusion tensor model was fitted for each voxel from which tensor-derived FA, MD, RD and AD maps were subsequently calculated (Fig 3). Diffusion characteristics of NAWM, and NAWM plus WMH (All_WM) were evaluated. Additionally, to quantify the stability of DTI parameters over time, MD histogram measures were computed in CSF spaces. Normalised histograms of 1000 bins were produced for FA, MD, RD and AD maps of the WM segmentations (FA range 0–1, bin width 0.001; MD, AD and RD range 0–0.004, bin width 4×10^−6^). Since FA and diffusivity measures are non-normally distributed in WM, we evaluated longitudinal change of the following histogram parameters: median, peak value, peak height, skew and kurtosis. These parameters have also been shown to have associations with cognition in a healthy older adult and SVD population [6,29,30].

#### 2.3.4 Missing MRI data

Out of the 99 patients included in the current study, for each of the consecutive three year follow ups, scanning sessions were performed on 98, 77 and 71 patients, with DTI respectively available for 99, 96, 74 and 68 patients (Fig 1). Disproportionate geometrical warping in diffusion-weighted images hindered accurate co-registration of four cases at baseline and two at year 1. Reasons given for withdrawal from the study are provided in Fig 1.

During follow-up three patients suffered new clinical strokes. Of these one patient had a small cortical haemorrhage which was considered a study endpoint. The other two patients had new small subcortical infarcts and were eligible to remain in the study according to the protocol, one was however unable to do so due to disability and withdrew.

### 2.4 Statistical analysis

Differences between baseline demographics and neuroimaging characteristics of the 99 patients included in the current study and those who did not attend any follow-up MRI were evaluated using Mann-Whitney U and χ^2^ tests where appropriate in Statistical Package for Social Sciences version 20.0 (SPSS, Chicago, IL, USA.).

Linear mixed-effects (LME) models in MLwiN (version 2.1, Centre for Multilevel Modelling, University of Bristol [31]) were used to assess the effects of time on the change in DTI parameters in All_WM and NAWM tissue segmentations. The trajectories of the DTI parameters for each individual were modelled as a linear trend across the follow-up period as a function of time (exact time since baseline assessment in years). Initial intercept and slope of each patient’s linear trajectory were allowed to vary with both fixed and random effects. Fixed effect variation was accounted for by time, and random effect variation allowed for remaining inter-individual differences. The average fixed effect slope of time represents the average annualised change rate for a parameter. The change rates of each DTI parameter were evaluated using a Wald test. A Bonferroni corrected significance level of p ≤0.0023 was used to correct for multiple comparisons (i.e. 21 comparisons). Since sensitivity of progression markers depends on a combination of significance and magnitude of change, as well as robustness of a model, residual errors were also calculated for all LME models. For comparison reasons, the change rates and residual errors are also expressed as a percentage of the average intercept, which represent the average baseline value.

Sample size estimations were carried out for all DTI parameters using longpower statistical package in R version 3.02 (www.R-project.org) [32]. For these estimations all available data was used and minimum sample sizes per treatment arm were calculated for a hypothetical 3-year trial with measurements taken at even yearly intervals. A balanced trial design was assumed. A range of percentage treatment effects was calculated assuming 80% power with α = 0.05.

DTI analysis produces many related parameters. To determine whether a more predictive score could be developed by combining these different measures, we also performed principal component analysis (PCA) using SPSS 20.0 to reduce the number of DTI parameters into one component that reflected the underlying variability in the data. PCA was applied to baseline DTI values. Component scores for subsequent time points were calculated using the component coefficients and standardised DTI values based on baseline values. To overcome the multicollinearity issue of highly correlated variables, we chose to exclude RD and AD parameters from the PCA as they can be considered as subparameters of MD. When all candidate variables were included in the PCA we observed problems with multicollinearity (correlation matrix determinant <0.0001). When we restricted variables to those that declined significantly over time in our primary analysis this problem was ameliorated. Principal components were retained if eigenvalues were greater than 1. Promax rotation was applied. Following PCA, LME models were also created for the principal component scores.

## Results

### 3.1 Demographics

Study cohort demographics and the volumetric measures of the various tissue class segmentations are shown in Table 1. Patients not included in the longitudinal study cohort were significantly older (p = 0.008), and had a lower score on the MMSE (p = 0.001). The mean age of the analysed longitudinal study cohort was 68.9 (SD = 10.0) years at baseline, and 75.0 (SD = 6.8) years for the patients in the non-longitudinal cohort. No differences were observed in baseline brain volume and other neuroimaging characteristics between these cohorts.

**Table 1:** Baseline characteristics of the longitudinal study cohort.

Demographics and neuroimaging characteristics	Longitudinal study cohort	Non-longitudinal study cohort	p-values
	N = 99	N = 22	
**Age (yrs)**	**68.9 ± 10.0**	**75.0 ± 6.8**	**0.008**
**Gender, % male**	66.7	54.5	0.283
**MMSE score**	**27.9 ± 2.4**	**25.7 ± 3.2**	**0.001**
**Rankin score**	1.1 ± 1.0	1.7 ± 1.5	0.084
**Hypertension (%)**	92 (92.9)	20 (90.9)	0.744
**Hypercholesterolaemia (%)**	85 (85.9)	19 (86.4)	0.951
**Diabetes mellitus (%)**	19 (19.2)	5 (22.7)	0.599
**Current smoker (%)**	21 (21.2)	2 (9.1)	0.510
**Brain Volume (ml)**	1038.0 ± 104.1	1103.0 ± 121.3	0.065
**WMH lesion load (%)**	3.6 ± 2.5	4.2 ± 4.8	0.868
**Lacunes of presumed vascular origin [range]**	4.2 [0–27]	3.9 [0–14]	0.659

Numbers represent means ± standard deviation or percentages. Hypertension was defined as systolic blood pressure >140 mmHg, diastolic >90 mmHg or being on antihypertensive treatment. Hypercholesterolaemia was defined as a serum total cholesterol >5.2 mmol/l or treatment with a statin. Abbreviations: MMSE = Mini Mental State Examination; WMH lesion load = White matter hyperintensity volume as percentage of total brain volume. Bold values differ significantly between groups at a level of p <0.05.

### 3.2 DTI change over time

There was a highly significant change in most DTI parameters for both NAWM and All_WM with p-values ≤0.001 (Table 2). FA and diffusivity histogram metrics changed consistently with a decrease in anisotropy and an increase in diffusivity, indicating a general increase in WM ultrastructural damage over time. The percentage annualised change of different DTI parameters ranged from 0.01% to 4.43% in All_WM tissue, and 0.15–5.96% in NAWM.

**Table 2:** DTI parameter progression values calculated through linear mixed effect models.

	All_WM					NAWM				
	Yearly rate of change (SD)	% annual change	Residual error	% residual error	χ^2^	Yearly rate of change (SD)	% annual change	Residual error	% residual error	χ^2^
**Fractional Anisotropy**										
Median	**-2.17×10**^**−3**^ **(4.05×10**^**−4**^**)**	**-0.74**	**4.76×10**^**−5**^	**0.016**	**28.67**	**-2.82×10**^**−3**^ **(4.85×10**^**−4**^**)**	**-0.95**	**5.82×10**^**−5**^	**0.020**	**33.82**
Peak height	3.27×10^−7^ (5.04×10^−6^)	0.01	1.00×10^−8^	<0.001	0.004	1.01×10^−5^ (5.10×10^−6^)	0.33	1.03×10^−8^	<0.001	4.647
Peak value	**-6.13×10**^**−3**^ **(1.65×10**^**−3**^**)**	**-2.25**	**6.08×10**^**−4**^	**0.223**	**13.86**	**-6.28×10**^**−3**^ **(1.68×10**^**−3**^**)**	**-2.26**	**7.15×10**^**−4**^	**0.257**	**13.99**
Skew	**0.010 (0.002)**	**1.46**	**1.17×10**^**−3**^	**0.171**	**22.65**	**0.016 (0.003)**	**2.34**	**1.33×10**^**−3**^	**0.195**	**35.19**
Kurtosis	0.013 (0.006)	2.49	8.28×10^−3^	1.586	5.239	**0.025 (0.007)**	**5.07**	**8.06×10**^**−3**^	**1.635**	**13.36**
**Mean Diffusivity**										
Median (mm^2^s^-1^)	**5.36×10**^**−6**^ **(5.22×10**^**−7**^**)**	**0.67**	**7.86×10**^**−11**^	**<0.001**	**105.3**	**3.42×10**^**−6**^ **(4.66×10**^**−7**^**)**	**0.43**	**7.91×10**^**−11**^	**<0.001**	**53.94**
Peak height	**-3.72×10**^**−4**^ **(3.12×10**^**−5**^**)**	**-2.44**	**2.68×10**^**−7**^	**0.002**	**141.7**	**-3.35×10**^**−4**^ **(3.57×10**^**−5**^**)**	**-2.05**	**3.63×10**^**−7**^	**0.002**	**87.94**
Peak value (mm^2^s^-1^)	**2.85×10**^**−6**^ **(6.13×10**^**−7**^**)**	**0.37**	**1.11×10**^**−10**^	**<0.001**	**21.60**	**1.97×10**^**−6**^ **(5.79×10**^**−7**^**)**	**0.26**	**1.32×10**^**−10**^	**<0.001**	**11.64**
Skew	0.017 (0.012)	0.68	0.053	2.120	2.592	0.029 (0.015)	0.99	0.083	2.833	3.966
Kurtosis	-0.304 (0.129)	2.00	6.484	42.658	5.610	-0.091 (0.187)	-0.45	13.84	68.440	0.237
**Radial Diffusivity**										
Median (mm^2^s^-1^)	**6.42×10**^**−6**^ **(1.25×10**^**−6**^**)**	**0.97**	**2.04×10**^**−10**^	**<0.001**	**26.53**	**4.92×10**^**−6**^ **(1.21×10**^**−6**^**)**	**0.75**	**2.02×10**^**−10**^	**<0.001**	**16.49**
Peak height	**-2.70×10**^**−4**^ **(2.24×10**^**−5**^**)**	**-2.15**	**1.85×10**^**−7**^	**<0.001**	**144.6**	**-2.25×10**^**−4**^ **(2.48×10**^**−5**^**)**	**-1.69**	**2.41×10**^**−7**^	**0.002**	**81.85**
Peak value (mm^2^s^-1^)	**4.34×10**^**−6**^ **(1.34×10**^**−6**^**)**	**0.68**	**3.10×10**^**−10**^	**<0.001**	**10.42**	**5.47×10**^**−6**^ **(1.37E-6)**	**0.86**	**3.05×10**^**−10**^	**<0.001**	**16.01**
Skew	**0.037 (0.010)**	**1.56**	**0.039**	**1.641**	**14.01**	**0.042 (0.012)**	**1.78**	**0.053**	**2.246**	**13.45**
Kurtosis	-0.114 (0.097)	0.95	3.676	30.633	1.399	0.023 (0.132)	0.16	6.943	48.299	0.031
**Axial Diffusivity**										
Median (mm^2^s^-1^)	**6.39×10**^**−6**^ **(5.96×10**^**−7**^**)**	**0.60**	**1.12×10**^**−10**^	**<0.001**	**114.8**	**3.01×10**^**−6**^ **(5.86×10**^**−7**^**)**	**0.29**	**1.23×10**^**−10**^	**<0.001**	**26.36**
Peak height	**-1.24×10**^**−4**^ **(1.24×10**^**−5**^**)**	**1.47**	**4.59×10**^**−8**^	**<0.001**	**99.92**	**-6.18×10**^**−5**^ **(1.31×10**^**−5**^**)**	**0.68**	**6.51×10**^**−8**^	**<0.001**	**22.36**
Peak value (mm^2^s^-1^)	**3.82×10**^**−6**^ **(1.15×10**^**−6**^**)**	**0.38**	**3.95×10**^**−10**^	**<0.001**	**11.01**	1.46×10^−6^ (9.43×10^−7^)	0.15	3.52×10^−10^	<0.001	2.383
Skew	**0.061 (0.007)**	**4.02**	**0.011**	**0.725**	**83.27**	**0.073 (0.008)**	**4.58**	**0.019**	**1.192**	**89.63**
Kurtosis	**0.230 (0.035)**	**4.43**	**0.494**	**9.515**	**42.45**	**0.379 (0.04)**	**5.96**	**0.778**	**12.235**	**73.19**
**Principal component score**	**0.129(0.016)**	**714**	**0.050**	**274**	**69.05**	**0.131(0.018)**	**431**	**0.059**	**194**	**55.24**

Yearly rates of change are defined as the mean estimates of the fixed effects from the linear mixed effect models with their standard deviation (SD). Percentages annual change and residual error are with respect to the average baseline value. Bold values have significant annualised change rates at a Bonferroni corrected level of p ≤0.0023.

**Fig 4:**
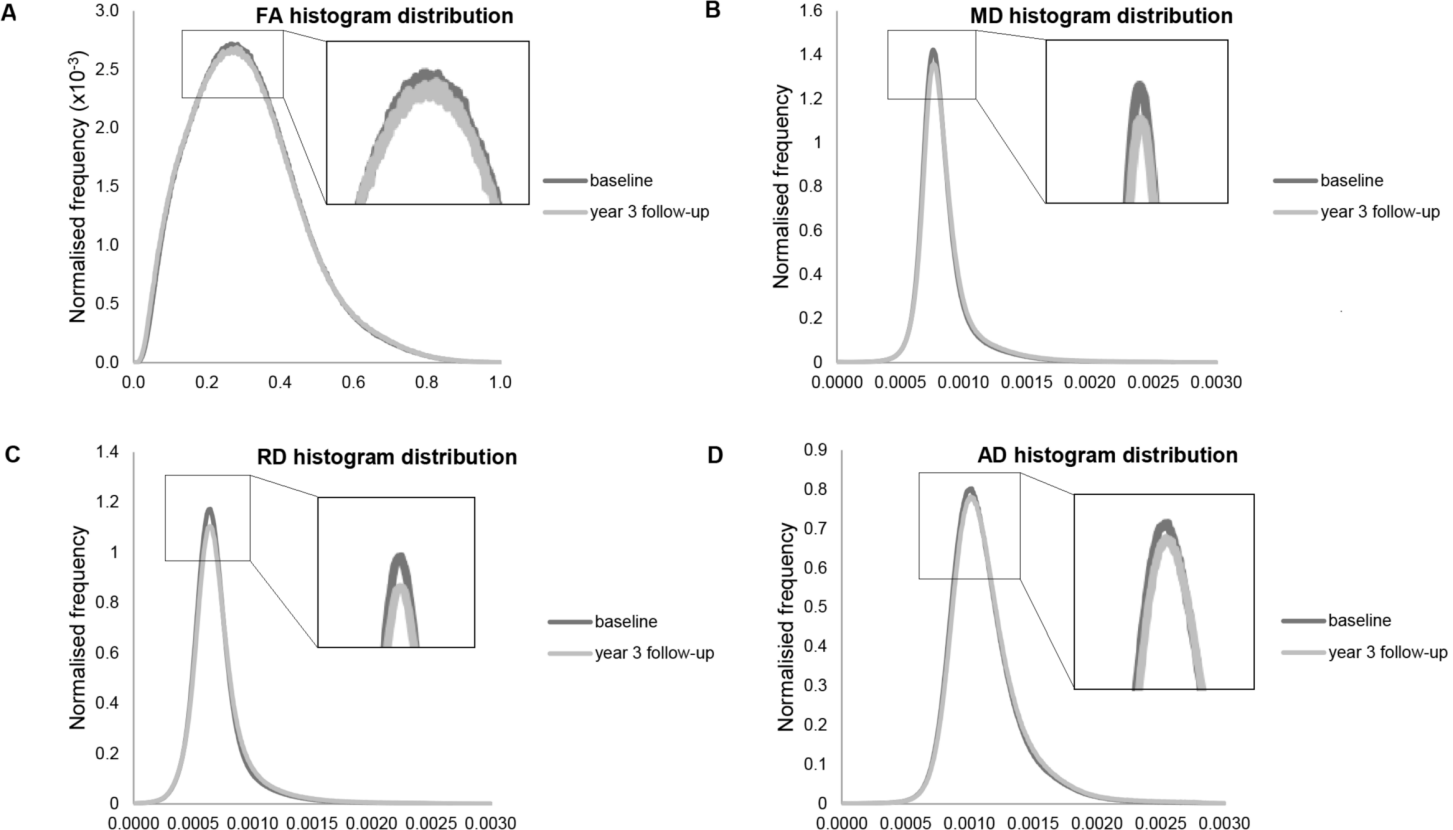
Average DTI histogram distributions of the baseline longitudinal cohort and at 3 year follow-up. A. Fractional anisotropy. B. Mean diffusivity (mm^2^s^-1^). C. Radial diffusivity (mm^2^s^-1^). D. Axial diffusivity (mm^2^s^-1^).

There was a change in the shape of the FA histograms over time due to a reduction in FA which manifested as decreased median and peak value, and an increased peak height (Fig 4A). This reduction in FA values also created a greater positive skew (i.e. longer tail to the right, All_WM: annualised change rate = 0.010, p <0.001; NAWM: annualised change rate = 0.016, p <0.001) and an increased kurtosis (i.e. a stronger peaked distribution, NAWM: annualised change rate = 0.025, p <0.001). Subtle differences over time were observed in the histogram distributions of the three diffusivity measures, MD, RD and AD (Fig 4B–4D). Increased diffusivity led to increased peak value and median, and decreased peak height values over time. Changes in skew and kurtosis were not significant for MD, but for RD and AD there was an increase in the skewness due to greater frequency of higher diffusivity voxels in the rightward tail (All_WM: annualised change rate RD skew = 0.037, p <0.001; annualised change rate AD skew = 0.061, p <0.001; NAWM: annualised change rate RD skew = 0.042, p <0.001; annualised change rate AD skew = 0.073, p <0.001). An increase in kurtosis was also found for the AD distribution representing a more peaked distribution (All_WM: annualised change rate = 0.230, p <0.001; NAWM: annualised change rate = 0.778, p <0.001).

A significant change of most DTI parameters was also observed within regions of WMH (S1 Table). The direction of change was, however, opposite to that observed in NAWM and All_WM. The estimated progression rates of FA metrics indicated increased anisotropy over time. The FA histogram showed an increased median and peak value, and decreased peak height (median FA: annualised change rate = 0.00332, p < 0.001; peak value FA: annualised change rate = 0.00648, p = 0.002; peak height FA: annualised change rate = -0.000171, p < 0.001). Changes in histogram parameters of MD, RD and AD within WMH over time indicated reduced diffusivity, as evidenced by decreased medians (MD: annualised change rate = -8.20×10^−6^ mm^2^s^-1^, p < 0.001; RD: annualised change rate = -7.64×10^−6^ mm^2^s^-1^, p < 0.001) and peak values (MD: annualised change rate = -1.46×10^−5^ mm^2^s^-1^, p < 0.001; RD: annualised change rate = -1.06×10^−5^ mm^2^s^-1^, p < 0.001; AD: annualised change rate = -1.08×10^−5^ mm^2^s^-1^, p = 0.002). Histogram peak height was reduced over time in all diffusivity measures and skew increased.

Within the CSF the MD median, peak height, peak value and kurtosis remained stable over time (see S2 Table). A significant change was found in MD skew in the CSF over time, such that the skew became less negative, indicating that the MD distribution within the CSF became more Gaussian over time (annualised change rate = 0.00267, p = 0.009).

Percentage residual error for each model was highly varied within both All_WM and NAWM. In particular kurtosis of the diffusivity measures had high residual errors: 9.515–42.658% within All_WM, and 12.235–68.440% within NAWM. Residual errors of skew models in the diffusivity DTI parameters were also relatively high. For MD and RD, these errors were larger than the annualised skew change rates.

The extracted principal component scores for All_WM and NAWM accounted respectively for 80.6% and 74.9% of the variance in the PCA. Very large annualised change rates were observed (annualised change All_WM: 714%; NAWM: 431%). Residual errors were equally large but did not exceed annualised change rates.

### 3.3 Sample size estimations

Table 3 shows the results of the sample size estimations. Sample sizes required for treatment trials varied greatly using the different DTI parameters. Assuming a treatment effect of 25% the minimum sample sizes needed per treatment arm for DTI parameters of All_WM ranged from 198 for MD peak height to 186043 for FA kurtosis, the range for NAWM being 356 for MD peak height to 3059376 for MD skew. The principal component scores had smaller sample size estimations than those of the majority of DTI parameters, with an estimation of 501 for the All_WM component score and 679 within NAWM if a treatment effect of 25% was observed. MD peak height however requires the smallest sample size.

**Table 3:** The minimum sample size estimation per arm required to test treatment effects of various percentages using DTI parameters.

	All_WM				NAWM			
	Minimum sample size (per arm) required to test treatment effects of				Minimum sample size (per arm) required to test treatment effects of			
	30%	25%	20%	15%	30%	25%	20%	15%
**Fractional Anisotropy**								
Median	1215	1749	2733	4859	945	1361	2126	3780
Peak height	4391	6323	9880	17565	194040	279417	436590	776159
Peak value	4461	6424	10038	17845	4354	6270	9798	17418
Skew	1779	2562	4003	7117	900	1295	2024	3598
Kurtosis	129197	186043	290694	516788	4935	7106	11103	19739
**Mean Diffusivity**								
Median	232	334	522	928	513	739	1154	2052
Peak height	138	198	310	551	248	356	557	990
Peak value	1869	2692	4205	7476	6193	8918	13935	24773
Skew	41083	59160	92437	164333	2124567	3059376	4780275	8498267
Kurtosis	66656	95982	149971	266615	7722	11120	17374	30888
**Radial Diffusivity**								
Median	1493	2150	3360	5973	3350	4825	7538	13402
Peak height	141	204	318	566	267	384	601	1068
Peak value	8787	12653	19770	35147	3485	5018	7841	13940
Skew	6023	8673	13551	24091	5875	8461	13220	23502
Kurtosis	34160	49190	76859	136639	3652	5259	8217	15607
**Axial Diffusivity**								
Median	183	264	412	733	1415	2038	3185	5662
Peak height	173	250	390	693	1799	2590	4047	7195
Peak value	7342	10572	16519	29368	88158	126948	198356	352632
Skew	268	386	604	1073	251	361	565	1004
Kurtosis	737	1062	1659	2950	337	485	758	1348
**Principal component score**	348	501	783	1392	472	679	1061	1887

## Discussion

Our results demonstrate that DTI is sensitive to change in patients with SVD over a relatively short duration of follow-up. Observed changes in DTI parameters were consistent with a progressive degradation of WM ultrastructure showing a decline in anisotropy and increase in diffusivity in WM. However our analysis demonstrates that different DTI parameters show marked differences in the sensitivity to change and errors. We have shown that the most sensitive marker for SVD progression is MD peak height. This finding indicates greater stability of MD values compared to its radial and axial counterparts, as well as sensitivity to change and a good model fit. We also provide sample size estimates, which will be useful in planning clinical trials using DTI as a surrogate marker. It follows that MD peak height has the smallest sample size estimate.

Over three year follow-up most DTI histogram parameters changed significantly. Interpretation of longitudinal DTI changes is not always straightforward. Changes in diffusivity histogram distributions are due to increased tissue ultrastructural damage that manifests as increases in diffusivity. This results in higher median and peak value, and a lower peak height as higher diffusivity values are identified with greater frequency. Increases in diffusivity values cause the rightward tail of the distribution to become broader, increasing the positive skew. Also, the related reduction of lower diffusivity values causes the diffusivity histograms to become less peaked and have lower kurtosis. This negative change is observed in MD and RD (All_WM), although it does not reach significance. For the remainder of kurtosis values, in RD (NAWM) and AD, kurtosis increases, significantly in the case of the latter. This finding most likely reflects the instability of AD as a reliable DTI measurement, indicated by the high residual errors in AD kurtosis models. Since AD is the diffusivity in the principal diffusion direction, errors in eigenvalue sorting and inaccuracies in the approximation of the principal diffusion direction using the DTI model will have a large impact on measurement of the AD parameter [33]. High residual errors in AD models therefore indicate limited value of this parameter for longitudinal assessment of SVD. MD, and to a lesser extent RD, may be more informative as they are calculated from the average of two or more eigenvalues and appear to be more stable measures.

Changes in the FA histogram distribution are due to reductions in FA that manifest as a lower median and peak value, and a higher peak height due to identification of lower FA values with greater frequency. In turn this causes a greater rightward tail of the distribution (greater positive skew) and a more pronounced peak (greater kurtosis). However, FA histogram parameters were found to have a poor longitudinal model fit with high residual error. This may be due to ambiguity of FA values. In particular, as FA is defined by a quadratic function in three related variables, similar FA values can represent different diffusion tensor (ellipsoidal) shapes [34]. The dependency of FA on the three eigenvalues of the diffusion tensor leads to reduced stability of FA compared to MD. The use of a component score combining MD and FA measurements inevitably decreases sensitivity to detection of disease progression compared to MD measurements alone. The observed sample size estimates of component scores also reflect this effect.

Samples sizes depend on the detected power of observations. Power in turn relies on error, so depends on measurement stability, effect size and in this case magnitude of change. Since scanner upgrades can affect measurement of DTI histogram parameters [35], their effect was evaluated. The results indicated that there was no significant change in MD histogram parameters in CSF spaces, apart from for MD skew. In this case the results indicated that the MD distribution was becoming more Gaussian over time, representing an improvement in MD quantification. These results assure validity of the observed rates of change in this study. The finding of lowest sample size estimates for MD peak height and median therefore demonstrates that the MD peak height benefits from being a highly stable measurement that is also sensitive to change. Whether DTI parameters are calculated for All_WM or NAWM also impacts sample size. Histologically WMH tissue shows axonal loss and glial proliferation [36]. DTI studies have shown that diffusivity is increased and anisotropy is lowered in WMHs compared to NAWM in SVD and CADASIL, a young-onset genetic variant of SVD [4,37]. This contributes to our finding of greater change in diffusivity parameters of All_WM tissue. Sample sizes estimates of All_WM diffusivity parameters are therefore reduced compared to parameter estimation in NAWM. For FA parameters, however, greatest change and lowest samples size estimates were obtained for FA measures from NAWM tissue compared with FA measures from All_WM. The severity of damage found in WMHs affects the uncertainty of the orientation of the principal diffusivities from which FA is calculated and will likely provide inconsistencies in FA [34]. This leads to greater sample size estimates for FA parameters within All_WM. Changes to diffusion characteristics of WMHs over time revealed the opposite effect to those observed in in NAWM and All_WM. The increase in anisotropy and decrease in diffusivity over time suggests improvement of tissue microstructure within WMHs; however, this is unlikely to be the case. Maillard et al., [38] have shown that voxels containing higher FA and lower diffusivity values are located at the edges of WMHs, indicating that the core of WMHs has greater microstructural damage than newly formed areas of WMH. Our observation of an apparent tissue microstructural improvement is likely to be the consequence of WMH growth (and changes in the surface area to volume ratio of the WMHs) and provides a misleading indication of improvement. Consequently, WMH diffusion characteristics provide an ambiguous and unsuitable marker for monitoring disease progression.

Despite relatively high patient dropout rates which could bias the sample to those with less decline, significant changes were detected in our cohort. The patient group who did not attend follow-up were older and had worse cognitive levels, indicating that such patients may be more vulnerable to decline and consequently an underestimation of actual change in DTI parameters in the study population. Secondly, the use of small histogram bin-widths in our analyses increased noise in measurements of peak value and peak height. A trade-off between sensitivity and noise is applicable when deciding on histogram bin size. We have however shown that annualized change rates of DTI parameter peak value and peak height are small and therefore require small histogram bin widths to enable detection of change. Also, we have generated histograms from WM tissue because SVD predominantly affects the subcortical structures, and in pilot studies we have shown that DTI parameters were sensitive to change in WM over short time periods in SVD [29]. However, it is now recognised that SVD does cause cortical GM pathology as well, with both atrophy and micro infarcts, and it is possible that a global whole brain DTI measure may be a simpler alternative. Finally, the absence of an age-matched control population is a limitation to this study, as observed changes in diffusion characteristics could be a result of SVD associated pathology and ageing. The ageing literature shows varying change rates of diffusion parameter. Most studies reveal smaller rates of change than found in our cohort. Nusbaum et al., [39] for example found age-related histogram changes in ADC, a diffusion weighted imaging equivalent to MD in SVD [35], that were approximately 8 times smaller than our MD changes. More recently, longitudinal FA and diffusivity changes in ageing have been shown to have annual percentage changes 2–3 times smaller than those in our cohort [40]. Lastly, a study directly comparing DTI parameters of CADASIL patients to age-matched controls revealed no change in MD peak value in the ageing population and an annual progression of 3.65×10^−6^ mm^2^s^-1^ in CADASIL [41] which is slightly greater than the 2.85 over time indicated reduced diffusivity, as evidenced by decreased medians 10^−6^ mm^2^s^-1^ change found in this study. Charlton et al., [30] on the other hand, reported slightly larger FA and MD changes in their elderly population. This discrepancy could be the result of quantifying change as the difference between two time points. Measurement noise and missing data will have a greater effect on change rates the fewer follow-up time points are acquired. Previously we have shown that analysis of fewer data points leads to greater magnitudes of change, as shown by smaller sample size estimates of hypothetical clinical trials [42]. Together these findings assure us that the progression rates as measured in our cohort represent SVD progression beyond pure age-related WM changes.

Our data suggests disease progression in SVD can be monitored sensitively using DTI histogram analysis. This is in line with earlier whole brain DTI histogram in ageing [30], sporadic SVD [29] and CADASIL [41], that have provided highly reproducible DTI markers suitable for longitudinal studies [43,44]. Since changes in cognition over the same duration in the same cohort have been small and significant for only one cognitive domain or even absent, depending on the statistical analysis applied [2,42], we suggest DTI could be attractive as a surrogate marker to follow disease progression and test the effect of therapeutic interventions in SVD. Other imaging parameters that are thought to be potential surrogate markers are brain atrophy, WMH growth and incident lacunes. Previously we have shown in the same cohort that development of new lacunes only occurred in a minority of SVD patients, there was however significant atrophy and WMH growth over a period of 3 years [42]. Additionally, in [42] we investigated progression of one DTI parameter, MD peak height of All_WM. Sample size estimates showed that WMH volume and MD peak height were most sensitive to change and most suitable as surrogate marker of disease progression. The current study provides evidence that MD peak height of All_WM is the most sensitive DTI parameter for use in longitudinal SVD studies.

Apart from being able to detect prospective change MRI markers will have to meet further requirements to fulfil all criteria of a validated surrogate marker [45]; one of the most important being, being able to predict future clinical cognitive decline. Such associations have been weak and inconsistent with regards to WMH volume [29,41,46]. Previous cross-sectional studies have on the other hand shown that DTI histogram parameters correlate strongly with cognition in SVD populations [6,8,29]. Analyses of longitudinal correlations between cognitive and DTI change, and investigating the interventional effect on DTI markers and cognition therefore now clearly represent the next phase for determining the potential of DTI as a future surrogate marker of SVD. The present study is continuing cognitive follow-up to 5 years, and plans to correlate cognitive changes over this period with MRI parameters if significant cognitive change is detectable over this longer period.

In summary, we observed significant longitudinal change in various DTI histogram parameters in WM in a SVD cohort of patients with symptomatic lacunar stroke and confluent areas of WMH of presumed vascular origin. The reliability and sensitivity of individual DTI histogram parameters as markers of disease progression is however highly variable. Our results suggest MD peak height is the most sensitive marker for detecting ultrastructural brain tissue damage in SVD. Future studies investigating progression of SVD and the use of DTI as a surrogate disease marker are needed and should focus on the relationship between the clinical outcomes of SVD and imaging markers.

## Supporting Information

S1 Dataset Minimal dataset underlying the presented findings. (XLS)

S1 Table Progression of DTI parameters within white matter hyperintensities calculated through linear mixed effect models. (DOCX)

S2 Table Progression of mean diffusivity values within CSF spaces. (DOCX)
