# Supplementary material for: Application of Diffusion Tensor Imaging Parameters to Detect Change in Longitudinal Studies in Cerebral Small Vessel Disease
Source: PLoS One. 2016 Jan 25;11(1):e0147836. doi: 10.1371/journal.pone.0147836 (PMC4726604; doi:10.1371/journal.pone.0147836)
Supplement: S1 Table — (DOCX) [file pone.0147836.s002.docx]

Yearly rates of change are defined as the mean estimates of the fixed effects from the linear mixed effect models with their standard deviation (SD). Percentages annual change and residual error are with respect to the average baseline value. Bold values have significant annualised change rates at a Bonferroni corrected level of p ≤0.0025.

|  | WMH |  |  |  |  |
| --- | --- | --- | --- | --- | --- |
|  | **Yearly rate of change (SD)** | **% annual change** | **Residual error** | **% residual error** | **χ^2^** |
|  |  |  |  |  |  |
| Fractional Anisotropy | |  |  |  |  |
| Median | **3.32E-3 (6.49E-4)** | **1.18** | **1.03E-4** | **0.037** | **26.15** |
| Peak height | **-1.71E-4 (1.99E-5)** | **-3.89** | **9.45E-8** | **0.002** | **73.31** |
| Peak value | **6.48E-3 (2.14E-3)** | **2.51** | **1.68E-3** | **0.65** | **9.15** |
| Skew | 0.010 (0.004) | 1.85 | 0.006 | 1.18 | 5.30 |
| Kurtosis | 0.033 (0.013) | 18.05 | 0.043 | 23.03 | 6.58 |
| Mean Diffusivity | |  |  |  |  |
| Median | **-8.20E-6 (1.82E-6)** | **-0.83** | **5.60E-10** | **<0.001** | **20.42** |
| Peak height | -1.68E-4 (6.79E-5) | -1.42 | 8.99E-7 | 0.007 | 6.13 |
| Peak value | **-1.46E-5 (2.69E-6)** | **-1.64** | **1.74E-9** | **<0.001** | **29.40** |
| Skew | **0.054 (0.013)** | **2.89** | **0.037** | **1.98** | **17.78** |
| Kurtosis | 0.158 (0.085) | 3.13 | 1.674 | 33.21 | 3.47 |
| Radial Diffusivity | |  |  |  |  |
| Median | **-7.64E-6 (2.22E-6)** | **-0.92** | **5.94E-10** | **<0.001** | **11.88** |
| Peak height | **-1.85E-4 (6.01E-5)** | **-1.85** | **6.54E-7** | **0.007** | **9.48** |
| Peak value | **-1.06E-5 (2.86E-6)** | **-1.45** | **1.37E-9** | **<0.001** | **13.65** |
| Skew | **0.060 (0.011)** | **3.62** | **0.029** | **1.75** | **29.39** |
| Kurtosis | 0.193 (0.064) | 4.83 | 0.844 | 21.09 | 9.11 |
| Axial Diffusivity |  |  |  |  |  |
| Median | -5.29E-6 (2.03E-6) | 0.40 | 7.58E-10 | <0.001 | 6.76 |
| Peak height | **-2.91E-4 (3.69E-5)** | **3.57** | **3.63E-7** | **0.004** | **63.32** |
| Peak value | **-1.08E-5 (3.43E-6)** | **0.86** | **3.54E-9** | **<0.001** | **9.82** |
| Skew | **0.064 (0.012)** | **4.78** | **0.047** | **35.07** | **28.06** |
| Kurtosis | 0.177 (0.062) | 5.33 | 1.103 | 33.22 | 8.15 |

**S1 Table. Progression of DTI parameters within white matter hyperintensities calculated through linear mixed effect models.**
