# Supplementary material for: Application of Diffusion Tensor Imaging Parameters to Detect Change in Longitudinal Studies in Cerebral Small Vessel Disease
Source: PLoS One. 2016 Jan 25;11(1):e0147836. doi: 10.1371/journal.pone.0147836 (PMC4726604; doi:10.1371/journal.pone.0147836)
Supplement: S2 Table — (DOCX) [file pone.0147836.s003.docx]

|  | CSF |  |  |  |  |  |
| --- | --- | --- | --- | --- | --- | --- |
|  | **Yearly rate of change (SD)** | **% annual change** | **Residual error** | **% residual error** | **χ^2^** | **p-value** |
|  |  |  |  |  |  |  |
| Mean Diffusivity | |  |  |  |  |  |
| Median | -6.69E-6 (6.78E-6) | 0.36 | 1.82E-8 | <0.001 | 0.98 | 0.322 |
| Peak height | -2.75E-5 (6.83E-5) | 1.02 | 2.05E-5 | 0.076 | 0.16 | 0.689 |
| Peak value | -6.55E-6 (2.27E-5) | 0.03 | 2.07E-7 | 0.009 | 0.08 | 0.777 |
| Skew | **0.0267 (0.010)** | **8.34** | **4.15E-2** | **12.97** | **6.90** | **0.009** |
| Kurtosis | -2.97E-2 (2.11E-2) | 4.56 | 0.197 | 30.21 | 1.98 | 0.159 |

**S2 Table. Progression of mean diffusivity values within CSFspaces**

Yearly rates of change are defined as the mean estimates of the fixed effects from the linear mixed effect models with their standard deviation (SD). Percentages annual change and residual error are with respect to the average baseline value. Bold values have significant annualised change rates at a level of p ≤0.05.
